# Everybody's Page

**Published:** 1891-02-21

**Authors:** 


### Everybody's Page.

The latest report of Dr. Sedgwick Saunders, public analyst of the City of London, is satisfactory in one respect at least. No adulteration has been found either in drugs or in groceries, but our old friend the milkman seems unable to overcome the fatal fascination of the pump, which continues, year by year, to tempt him from the path of virtue in a more or less degree. What grieves Dr. Saunders apparently more than the actual adulteration of food which takes place annually is the apathy of the public, who show an exasperating indifference to the possession of the powers placed in their hands by the various Acts of Parliament, which thus become practically inoperative.

Whatever may be the ultimate result of "General" Booth's latest scheme, which has aroused so much diversity of opinion, its discussion appears to have done indirect good already. The Guardians of the City of London Union have taken into consideration the desirability of approaching other Boards of Guardians with the view of obtaining, through the medium of the Local Government Board, improvements in the whole system of accommodation for "casuals," and of discussing the question of establishing inebriate homes for the poor. The desirability of isolating this latter class, whose presence amongst their fellow-beings is a constant source of corruption, will probably be unquestioned by most people: the *crux* will be the cost to the ratepayer.

The comprehensive report of the Royal Commission on the Blind, &c., will be found more instructive reading than most blue books by those who have the patience to wade through fifty folio pages. The description by the Commissioners of Gardner's Trust for the Blind, as of the greatest service in assisting the blind to help themselves, is one which other societies might well strive to deserve. What is principally needed is to make the blind more independent of charitable aid than they now are, to show more discrimination in the istribution of pensions which, with all classes of society prove o often a discouragement to industry, and to base their education on such a principle as to lessen the isolation which cuts them off from the rest of the world and to deepen their connection with their more fortunate fellow beings.

Dr. P. Moreau, of Tours, gives some very curious information in the *Annales Médico-Psychologiques* as to suicides and the curious and different forms the mania takes in different countries. In Europe generally strangulation seems to have the greatest fascination for the unfortunate people who contemplate life as not worth living; next comes suicide by drowning; and then by shooting. It appears that there is a stronger propensity amongst the French and Italians, than amongst the inhabitants of any other country to throw themselves from a height. Poisoning is of most frequent occurrence in this country, more especially in Ireland, being rare in other countries, with the exception of Italy; and France is distinguished by the exceptional number of deaths from asphyxiation caused by the fumes arising from coke. Speaking generally, men appear to hang themselves and cut their throats oftener than women, who prefer poisoning, asphyxiation, and throwing themselves from a height.

Hippocrates says: "The second best remedy is better than the best, if the patient likes it best." We fully agree with Hippocrates.

A record of many of the curious things people have done in fits of absent-mindedness would probably prove an amusing and instructive compilation. To forget that you have a dinner party and guests to entertain, and to go to bed when you should be dressing for dinner is not altogether desirable in the eyes of those who have good appetites and look forward momentarily to their indulgence. Topham Beauclerk, a well-known character in his day, is stated to have been guilty of this misdemeanour, and was found fast asleep by his servant.

A good deal of difference of opinion seems to have been expressed at a recent meeting convened in West Southwark, as to the amount of distress now prevalent. No doubt it is at all times difficult to determine with great accuracy the amount of genuine distress, for statistics are at the best but doubtful guides. Many of the greatest sufferers are averse to publishing their woes, and of such statistics must necessarily be silent. It was stated by one of the St. Saviour's guardians that in 1870 10,000 cases were on the books, whereas at the present time they had less than 1,000. This certainly would seem to point to the state of the labour market being very much more favourable than one has been led to suppose.

An instructive instance of the simplicity which is such a charm in the character of the Heathen Chinee is given in a report of Dr. Daly, physician in charge of the Ningpo Missionary Hospital. This gentleman observes that Chinese teeth are more easily extracted than Europeans'. Lucky Chinamen! This fact will cause no surprise when we learn that, according to the natives, the dentists possess a powder with almost magic properties. The procedure is as follows: The powder is rubbed on the gum, after an interval of about five minutes the patient is told to sneeze, and the tooth falls out! What could be more simple? And Dr. Daly is unkind enough to cast a slur on the guilelessness of Chinamen by challenging them to extract a tooth in this manner in his presence, but they are too astute to accept the challenge.

That much-desired end, ventilation without a draught, seem to have been at last accomplished, and will prove a great blessing in all public buildings. The Hoey system, which effects the inlet ventilation by means of fresh air reservoirs, in which are enclosed ranges of hot-water pipes if desired, and the passing away of the vitated air through an opening in the wall at the level of the ceiling, must have proved an inestimable blessing to those who have had to suffer for so long the draughts which gave the Law Courts an unenviable notoriety. The outflow of air can be regulated by a wire gauze moveable screen or shutter, and the whole atmosphere in a large building can be renewed completely as often as desired without the slightest draught or current being felt. It is even stated, that during the recent dense fogs the atmosphere in Messrs. Barclay's bank was thus kept quite clear.

